# Identification of zinc finger protein Bcl6 as a novel regulator of early adipose commitment

**DOI:** 10.1098/rsob.160065

**Published:** 2016-06-01

**Authors:** Xiaoming Hu, Yuanfei Zhou, Yang Yang, Jie Peng, Tongxing Song, Tao Xu, Hongkui Wei, Siwen Jiang, Jian Peng

**Affiliations:** 1 Department of Animal Nutrition and Feed Science, Breeding and Reproduction of Ministry of Education, College of Animal Science and Technology, Huazhong Agricultural University, Wuhan 430070, People's Republic of China; 2 Key Laboratory of Animal Genetics, Breeding and Reproduction of Ministry of Education, College of Animal Science and Technology, Huazhong Agricultural University, Wuhan 430070, People's Republic of China; 3 The Cooperative Innovation Center for Sustainable Pig Production, Wuhan 430070, People's Republic of China

**Keywords:** adipogenesis, Bcl6, STAT1, transcription regulation, zinc finger proteins

## Abstract

Adipose tissue is a key determinant of whole-body metabolism and energy homeostasis. Unravelling the transcriptional regulatory process during adipogenesis is therefore highly relevant from a biomedical perspective. In these studies, zinc finger protein B-cell lymphoma 6 (Bcl6) was demonstrated to have a role in early adipogenesis of mesenchymal stem cells. Bcl6 is enriched in preadipose versus non-preadipose fibroblasts and shows upregulated expression in the early stage of adipogenesis. Gain- and loss-of-function studies revealed that Bcl6 acts as a key regulator of adipose commitment and differentiation both *in vitro* and *ex vivo*. RNAi-mediated knockdown of *Bcl6* in C3H10T1/2 cells greatly inhibited adipogenic potential, whereas *Bcl6* overexpression enhanced adipogenic differentiation. This transcription factor also directly or indirectly targets and controls the expression of some early and late adipogenic regulators (i.e. Zfp423, Zfp467, KLF15, C/EBPδ, C/EBPα and PPARγ). We further identified that Bcl6 transactivated the signal transducers and activators of transcription 1 (*STAT1*), which was determined as a required factor for adipogenesis. Moreover, overexpression of *STAT1* rescued the impairment of adipogenic commitment and differentiation induced by *Bcl6* knockdown in C3H10T1/2 cells, thereby confirming that *STAT1* is a downstream direct target of Bcl6. This study identifies Bcl6 as a positive transcriptional regulator of early adipose commitment.

## 1. Introduction

Obesity is a serious health problem worldwide caused by a chronic imbalance between energy expenditure and energy storage by adipose tissue and often promotes a range of chronic diseases, including atherosclerosis, hypertension and type 2 diabetes [1]. Adipocytes are the main constituent of adipose tissue and are considered to be a cornerstone in the homeostatic control of whole-body metabolism [2]. Adipogenesis, which plays a key role in the hyperplasia of the fat cell, is defined as a two-step developmental process in which an undifferentiated mesenchymal stem cell (MSC) commits into a preadipocyte, which then undergoes a terminal differentiation step to become a lipid-filled adipocyte [3,4].

Over the past two decades, the transcriptional networks controlling the process of terminal adipocyte differentiation have been extensively studied in committed preadipocyte cell lines, such as 3T3-L1 or 3T3-F442A [5,6]. This process is orchestrated by a transcriptional cascade involving the nuclear receptor peroxisome proliferator activated receptor-γ (PPARγ) and members of the CCAAT/enhancer-binding proteins (C/EBPs) family [7]. PPARγ in particular is considered the master regulator of adipogenesis [7]. Recent efforts have focused on determining molecular factors that define the committed preadipocyte phenotype. Some transcriptional components, such as zinc finger protein (Zfp) 423 [8], pRb [9], Zfp467 [10], TCF7L1 [11], Zfp521 [12], Zfp395 [13] and ZEB1 [14], were identified as early regulators during adipogenesis, thereby suggesting that other factors that may be involved in specifying adipogenic competency and commitment of MSCs remain to be discovered.

To elucidate other components of the adipogenic regulatory network, we performed a transcription factor (TF) screen based on our previous RNA-sequencing (RNA-seq) data during early adipogenesis. The data showed that the expression of 66 TFs was significantly upregulated at day 2 during the adipogenesis of porcine adipose or muscle stromal vascular (SV) cells [15]. Furthermore, through bioinformatics analysis and qPCR validation, we identified eight TFs which were upregulated during the early adipogenesis of SV cells and also have potential binding sites on the promoter of well-known pro-adipogenic factors, such as Zfp423, Zfp467, Ebf1 and ZEB1. Furthermore, we found that RNAi-mediated knockdown of *Bcl6* markedly inhibited adipogenic phenotypes of porcine SV cells (X Hu, Y Zhou, Y Yang 2014, unpublished data). Given the highly conserved domains of Bcl6 in vertebrates, this gene appears to be an excellent candidate factor that regulates critical early events during adipogenesis.

B-cell lymphoma 6 (Bcl6) is a Zfp that belongs to the POZ/ BTB family and is a transcriptional factor that was originally identified as a proto-oncogene [16]. The structure of the Bcl6 protein includes six Krüppel-type C-terminal zinc finger (ZF) motifs, a central PEST domain and an N-terminal POZ/BTB motif [17]. Studies have shown that Bcl6 is required for normal germinal centre (GC) development and is expressed at high levels in GC B cells and a subset of diffuse large B-cell lymphomas [18,19]. Recent evidence also indicated that Bcl6 is a critical regulator of bone development [20,21], and is involved in the adipose development through regulating lipid metabolism [22]. However, to date, there has been no evidence regarding the functional role of Bcl6 in adipocyte lineage commitment and adipogenesis.

Here, we identify such a role for Bcl6 as a positive regulator of adipogenesis and adipose tissue development. The expression of Bcl6 is enriched in preadipocytes and upregulated during the early stage of MSC adipogenesis and adipose development. The gain- and loss-of-function studies *in vitro* and *ex vivo* demonstrated that Bcl6 is a positive regulator of adipogenesis. This TF could directly or indirectly target and control the expression of some early and late adipogenic genes, such as *Zfp423*, *Zfp467*, *KLF15*, *C/EBPδ*, *C/EBPα* and *PPARγ*. We also identify signal transducers and activators of transcription 1 (*STAT1*) as a critical direct downstream target of Bcl6, and we show that STAT1 is required for adipogenesis and can rescue the adipogenic competency in *Bcl6* knockdown C3H10T1/2 cells. Our data establish that Bcl6 acts early in adipogenesis to positively regulate adipogenic commitment, at least in part through direct promotion of *STAT1* and indirect enhancement of *KLF15 and Zfp467* expression.

## 2. Material and methods

### 2.1. Cell culture and differentiation

NIH-3T3, C2C12, 3T3-L1 and C3H10T1/2 cells (American Type Culture Collection) were cultured in DMEM (Gibco) supplemented with 10% fetal bovine serum (FBS; Gibco) and 1% penicillin/streptomycin (Mediatech). For adipogenic differentiation, 2-day postconfluent (designated day 0) C3H10T1/2 cells were treated with 1 µM dexamethasone, 0.5 mM isobutyl-methylxanthine, 10 µg ml^−1^ insulin and 200 µM indomethacin (MDII). After 2 days, the cells were refed with 10% FBS medium containing only 10 µg ml^−1^ insulin for 2 days, then maintained in 10% FBS for 4 days. Neutral lipid accumulation at day 8 was assessed by oil red-O or BODIPY 493/503 staining.

### 2.2. Plasmids

*Bcl6* and *STAT1* cDNA were generated from a mouse cDNA library and cloned into the pCDNA3.1 vector (Invitrogen, USA) at the *HindIII* and *BamHI* sites, respectively. To construct the plasmids of pCMV-HA-Bcl6 CDS, pCMV-HA-Bcl6 ΔBTB, pCMV-HA-Bcl6 ΔPEST and pCMV-HA-Bcl6 ΔZF, the sequences of full-length Bcl6 and with deletion of the BTB domain, PEST domain or ZF domain were amplified and cloned into pCMV-HA (Clontech) vector at the *BglII* and *KpnI* sites. The primers used for PCR amplification are listed in the electronic supplementary material, table S1. PPARγ expression vector (pCMV-PPARγ) was saved in our laboratory [23]. A series of different *STAT1* promoter fragments were amplified by PCR from genomic DNA, sequence verified and cloned into the pGL3-basic vector (Promega) at the *KpnI* and *NheI* sites. For *STAT1*, the following five promoter constructs were generated: *STAT1* (−2031/+76), *STAT1* (−1645/+76), *STAT1* (−1288/+76), *STAT1* (−836/+76), and *STAT1* (−227/+76). Mutation and deletion of the postulated Bcl6 binding sequences within the mouse *STAT1/P1* (−2031/+76) and *STAT1/P2* (−1645/+76) promoter were done using the QuickChange^®^ II site-directed mutagenesis kit (Agilent Technologies, Germany) according to the manufacturer's recommendation. The primers are given in the electronic supplementary material, table S2.

### 2.3. RNA isolation and quantitative real-time PCR

Total RNA was extracted from tissue and cell samples using the Trizol reagent (Invitrogen) and treated with RNase-free DNase (MBI Fermentas, Germany), and then reversely transcribed using ReverTra Ace qPCR RT Kit (TOYOBO, Japan) according to the manufacturer's instructions. Quantitation of mRNA level by qPCR was performed using a real-time PCR System (Roche LC480, USA) by using iTaq Universal SYBR Green Supermix (Bio-Rad, USA). The primers used for qPCR are listed in the electronic supplementary material, table S3. The qPCR profile was 95°C for 2 min for enzyme activation, followed by denaturing at 95°C for 10 s, and annealing for 10 s and elongation at 72°C for 20 s, repeated for a total of 40 cycles. Data evaluation was performed using the LightCycler data analysis software (v. 3.5). All PCR amplifications were performed in triplicate for each RNA sample and gene expression levels were quantified relative to β-actin expression using LightCycler v. 480 Software. The results were analysed by the mode of 2^−ΔΔCt^.

### 2.4. RNA interference

Synthetic siRNA oligonucleotides specific for regions in the mouse *Bcl6* and *STAT1* mRNA were designed and synthesized by GenePharma (Shanghai, China). The sequences for successful knockdown were as follows: *Bcl6* siRNA-1, 5′-GCAGACGCACAGTGACAAA-3′; *Bcl6* siRNA-2, 5′-TGATGTTCTTCTCAACCTTAA-3′; *STAT1* siRNA-1: 5′-GACCCTAGAAGAATTACAA-3′; *STAT1* siRNA-2, 5′-GCTGAACT-ATAACTTGAAA-3′; *STAT1* siRNA-3, 5′-TGAGTTCCGACACCTGCAACTGAA-3′. *PPARγ* siRNA: 5′-CAACAGG-CCTCATGAAGAA-3′ [24]. Negative control (NC) siRNA was: 5′-TTCTCC-GAACGTGTCACGT-3′. C3H10T1/2 cells were transfected at 50–70% confluence with siRNA duplexes using Lipofectamine RNAi MAX (Invitrogen) according to the manufacturer's instructions. The coding sequences for *Bcl6* shRNA (the target sequence is siRNA-1 which has higher efficiency for reducing Bcl6 than siRNA-2) and NC shRNA (electronic supplementary material, table S4) were cloned downstream of the U6 promoter into the pRNAT-U6.1 plasmid according to the instructions from Ambion.

### 2.5. Oil red-O and BODIPY 493/503 staining

Lipid accumulation in adipocytes was assessed by oil red-O (Sigma-Aldrich, USA) or BODIPY 493/503 (D-3922, Thermo Scientific, USA) staining. Cells were washed three times with phosphate-buffered saline (PBS), followed by fixation with 4% paraformaldehyde in phosphate buffer for 1 h at room temperature. After fixation, the cells were washed again with PBS and stained with freshly diluted oil red-O (six parts oil red-O stock solution and four parts distilled H_2_O; the stock solution was 0.5% oil red-O in isopropanol) for 15 min, or stained with freshly diluted BODIPY 493/503 (10 µl of 1 mg ml^−1^ BODIPY 493/503 stock solution was added to 10 ml of 150 mM NaCl; the stock solution was prepared by dissolving 5 mg BODIPY 493/503 in 5 ml ethanol). Excess stain was removed using a small transfer pipette and four to five washings with distilled water.

### 2.6. Triglyceride content assays

The concentrations of triglycerides in the lysates of adipocytes were measured with commercial kits (Applygen Technologies, Beijing, China) following the manufacturer's instructions. The concentrations of the triglycerides were normalized to the protein content (μmol mg^–1^ protein) using a bicinchoninic acid (BCA) assay kit (Thermo Scientific Pierce Chemical, Rockford, IL, USA).

### 2.7. Western blotting

Protein samples were extracted with protein lysis buffer (10 mM HEPES (pH 7.6), 1.5 mM MgCl_2_, 0.5 mM DTT, 10 mM KCl, 10 mM NaF, 1 mM Na_3_VO_4_ and 0.5 mM PMSF) supplemented with protease inhibitor cocktail according to the manufacturer's protocol. The membranes were incubated with the following primary antibodies: anti-Bcl6 (ab19011, Abcam; D65C10, Cell Signaling Technology), anti-STAT1 (9172, Cell Signaling Technology), anti-PPARγ (AF6284, Affinity Biosciences), anti-adiponectin (2789S, Cell Signaling Technology), anti-HA (C29F4, Cell Signaling Technology) and anti-β-actin (sc-69879, Santa Cruz Biotechnology). Anti-mouse or anti-rabbit IgG-HRP (Invitrogen) was used to detect primary antibodies. The proteins were visualized using the SuperSignal chemiluminescence detection kit (Thermo Scientific) according to the manufacturer's protocol. Enhanced chemiluminescence signals were scanned using a FluorChem M apparatus (CareStream 2200 PRO, USA). The density of the bands was analysed using image analysis software (ImageJ).

### 2.8. Stable cell lines

For stable and selected lines, C3H10T1/2 cells at 80% confluence were transduced with the plasmids expressing shBcl6, shNC, Bcl6 or empty vector in 6-well plates. After 24 h, the cells were trypsinized and transferred into larger dishes and allowed to recover for 1 day prior to selection with 600 ng ml^–1^ G418 (Sigma, USA). After every 2 days, G418 selection medium was changed and the stably transduced cells were selected for two weeks. Subsequently, individual clones were isolated and grown separately in the presence of 360 ng ml^–1^ G418. Cell lysates were then assayed for identifying the Bcl6 mRNA and protein expression before performing actual experiments. The colony that reduced or overexpressed the expression of Bcl6 by the greatest amount was designated Bcl6 KD C3H10T1/2 or Bcl6 OE C3H10T1/2.

### 2.9. RNA extraction and RNA sequencing

The total RNA from the C3H10T1/2 stable cells of Bcl6 KD and Bcl6 OE was extracted using the Trizol reagent (Invitrogen) according to the manufacturer's instructions. The integrity and quality of the total RNA was checked using a NanoDrop 1000 spectrophotometer and formaldehyde agarose gel electrophoresis. RNA was only used when the Abs260 nm/Abs280 nm ratio was more than 1.8. For Illumina sequencing, the RNA samples from three independent biological replicates in each group were pooled with the same amount of total RNA. For RNA-seq library synthesis, 1 µg of total RNA was first depleted of rRNA using the Ribo-Zero rRNA Magnetic Kit (Plant Seed/Root kit, Epicentre, Madison, USA). Sequencing libraries were generated using the TruSeq RNA Sample Prep Kit (Illumina, Scoresby, Australia). Sequencing was then performed on a Hiseq 2500 as a 50 bp single-end run according to the manufacturer's instructions (Illumina, Scoresby, Australia).

### 2.10. RNA-seq analysis

Raw reads from each sequencing library were firstly cleaned using FASTX-Toolkit suite (http://hannonlab.cshl.edu/fastx_toolkit/) to remove adaptor sequences, reads with unknown sequences ‘N’ and low-quality sequences (the percentage of low-quality bases with a Phred quality score less than 20 was greater than 50% in a read). The clean reads were aligned to Ensembl 70 gene annotation of the NCBI38/mm10 genome using Bowtie with default parameters. The number of annotated clean reads for each gene was calculated and normalized to reads per kilobase per million (RPKM) [25]. Expression differences between the samples were quantified with DESeq [26]. The ‘false discovery rate (FDR) ≤ 0.05 and the value of shBcl6/shNC >1.5 or <0.7 and Bcl6/control >1.5 or <0.7′ were set as thresholds to judge the significance of gene expression difference. Gene ontology (GO) analysis was performed to further understand the biological functions of the genes within coordinate expression using the online bioinformatics database DAVID [27]. Significant GO categories with *p* < 0.05 were selected.

### 2.11. Chromatin immunoprecipitation

Chromatin immunoprecipitation (ChIP) assays were performed using a ChIP assay kit (Pierce, Thermo Scientific, Rockford, IL, USA), following the instructions of the supplier. Chromatin samples were prepared from WT and Bcl6 KD C3H10T1/2 stably selected cells at day 2 after MDII induction and then fixed with 1% formaldehyde, washed, and harvested in SDS lysis buffer. After sonication digestion, lysates containing soluble chromatin were incubated overnight with 4 µg of rabbit anti-Bcl6 antibody (D65C10, Cell Signaling Technology, USA) or rabbit IgG (Invitrogen). DNA–protein immunocomplexes were precipitated with salmon sperm DNA/protein-A–agarose beads, washed and eluted. The protein–DNA cross-links were reversed by treatment with proteinase K. Input control and DNA obtained from the immunoprecipitation were subsequently purified and used as templates to PCR-amplify mouse *STAT1*-specific sequences and for verification of ChIP enrichment by qPCR. The primers used for the amplification of the fragment spanning the putative Bcl6 binding site included: Fw: 5′-TACCTCTGCCTGCTTAGTA-3′, Rev: 5′-GCCAACATCTGTATTCTCAA-3′.

### 2.12. Transfection and luciferase reporter assays

C3H10T1/2 cells were plated in a 48-well plate and transfected with the luciferase reporter plasmids using Lipofectamine 2000 reagent (Invitrogen). For luciferase assays of the *STAT1* promoter constructs, 200 ng of promoter constructs and 200 ng of Bcl6 overexpression vector pCDNA3.1-Bcl6 or empty vector, and 20 ng of pRL-TK were co-transfected in C3H10T1/2 cells. Forty-eight hours after transfection, the cells were washed three times with cold PBS and cell lysates were prepared using Dual-Glo luciferase reagent (Promega, USA). The luciferase activity in 10 µl lysate was determined using a Dual-luciferase reporter assay system and luminometer (Dynex Technologies, UK). Transfection efficiency was normalized by *Renilla* luciferase activity measured concurrently in the same lysate. Transfections were performed in triplicate for each independent experiment.

### 2.13. Statistical analysis

Values are expressed as mean ± s.d. of at least three independent experiments. Prism was used to evaluate the data for statistical significance by two-tailed Student's *t*-tests (figures 2*i*,*j*,*l*, 3, 5 and 6*g*–*i*) and one-way ANOVA (figures 2*a*,*d*,*e* and 6*a*,*d*) with *p < 0.05 (versus the indicated controls) considered as significant.

## 3. Results

### 3.1. Bcl6 is enriched in preadipocytes and upregulated during early stage of MSC adipogenesis

**Figure 1.**
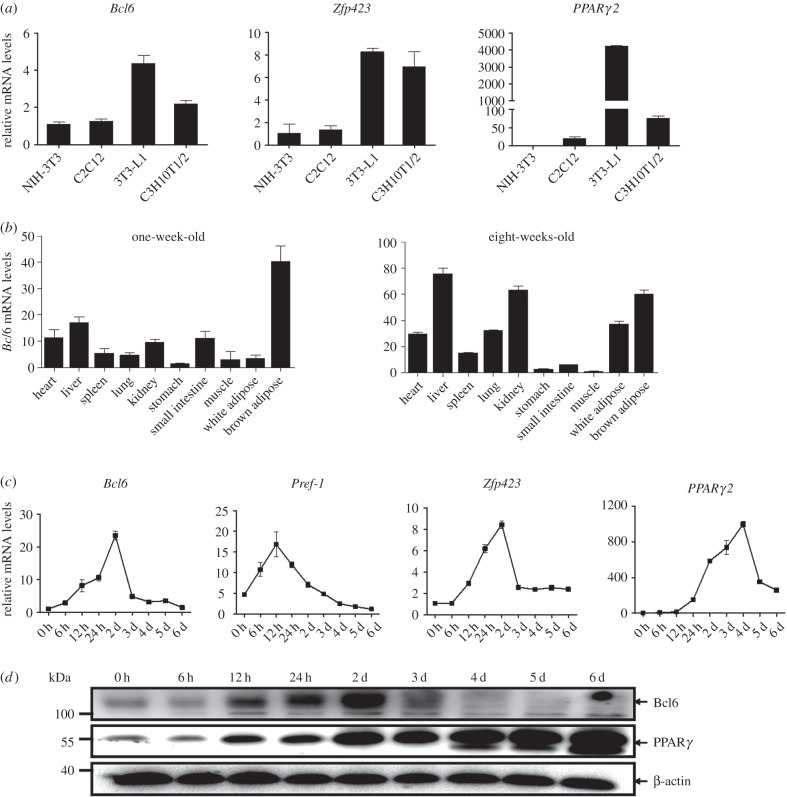
Bcl6 is enriched in preadipocytes and upregulated during early stage of MSC adipogenesis. (*a*) *Bcl6* expression in non-adipogenic NIH-3T3 and C2C12 fibroblasts, and adipogenic 3T3-L1 and C3H10T1/2 fibroblasts. Results are expressed as mean ± s.d. (*n* = 3). (*b*) Tissue expression pattern of the *Bcl6* gene in postnatal mice after one week and eight weeks. Tissue types are: heart, liver, spleen, lung, kidney, stomach, small intestine, muscle, white adipose tissue, brown adipose tissue. Results are expressed as mean ± s.d. (*n* = 3). (*c*) C3H10T1/2 cells were differentiated and RNA isolated at the indicated time points. Expression patterns of *Bcl6* and well-characterized initiators *Pref-1, Zfp423* and *PPARγ2* mRNA were measured by qPCR and normalized to β-actin. Results are expressed as mean ± s.d. (*n* = 3). (*d*) C3H10T1/2 cells were differentiated and protein sampled at the indicated time points. The expression of Bcl6 and PPARγ protein was measured by western blot.

Our previous study showed that expression of *Bcl6* was significantly upregulated at day 2 during the adipogenesis of porcine adipose SV cells [15], indicating its potential role in adipogenesis. To confirm the expression profile of *Bcl6*, we first evaluated the expression of *Bcl6* in the non-adipogenic NIH-3T3 and C2C12 fibroblasts, as well as the adipogenic 3T3-L1 and C3H10T1/2 fibroblasts. The results showed that in non-adipogenic cells there was low expression of *Bcl6*. Relatively, *Bcl6* mRNA was highly expressed in the 3T3-L1 preadipocytes, which have the greatest adipogenic potential in response to the standard hormonal cocktail when compared with the non-adipogenic fibroblasts; *Bcl6* mRNA was also expressed at moderate levels in multipotent C3H10T1/2 cells. This pattern was similar to the well-characterized initiator *Zfp423*, the key regulator of preadipocyte commitment (figure 1*a*). In mice, *Bcl6* mRNA levels were enriched in brown adipose tissue in postnatal mice after one week, whereas high expression was noted in white and brown adipose tissue after eight weeks, as well as high expression being observed in the liver and kidney where Bcl6 has numerous functions (figure 1*b*).

As anticipated, we found that the mRNA expression of *Bcl6* was upregulated during the early stage of adipogenesis in multipotent C3H10T1/2 cells, which was also similar to the expression pattern of *Zfp423*, but expressed earlier than *PPARγ2* (figure 1*c*). The protein expression pattern during adipogenesis of C3H10T1/2 cells was also significantly upregulated in the early stage, with the highest expression at day 2 detected by western blot analysis (figure 1*d*). These results indicate that Bcl6 is enriched in preadipocytes and adipose tissue, and its expression is upregulated during the early stage of MSCs adipogenesis.

### 3.2. Bcl6 is a positive regulator of early adipogenesis

**Figure 2.**
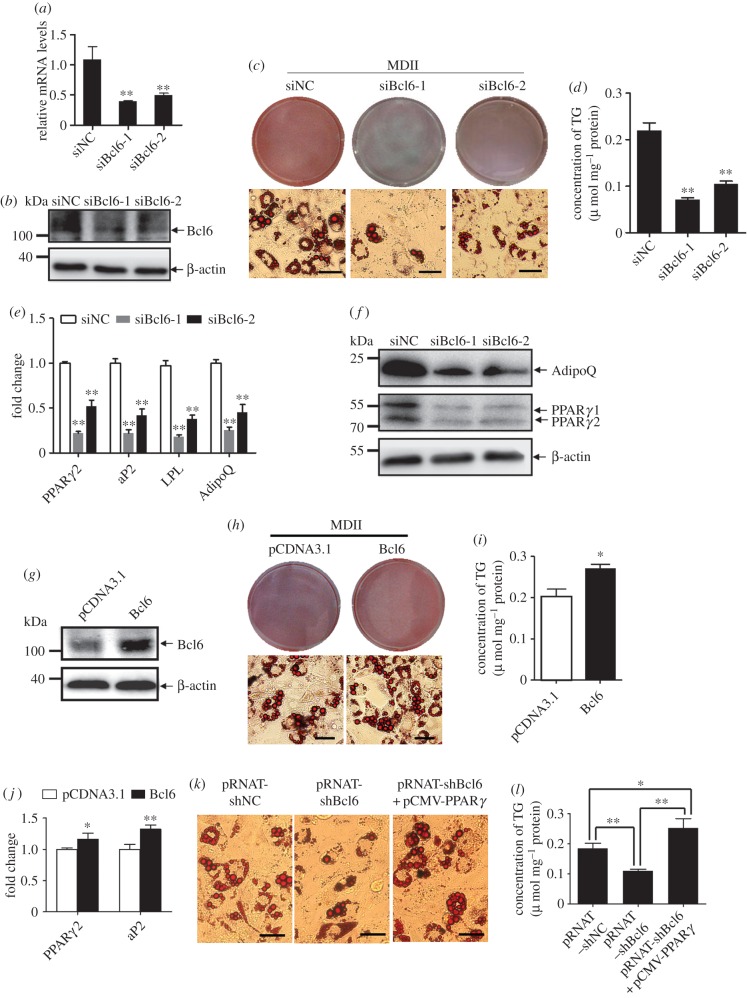
Bcl6 is a positive regulator of adipogenesis *in vitro*. (*a*,*b*) The *Bcl6* mRNA and protein expression levels after transfected siNC or siBcl6 after 36 h and 48 h, respectively. (*c*) Adipogenic phenotypes of C3H10T1/2 cells transiently transfected with synthetic siRNAs targeting *Bcl6* or NC siRNA after being induced for 8 days with MDII (0.5 mM 3-isobutyl-1-methylxanthine, 1 µM dexamethasone, 10 µg ml^−1^ insulin and 200 µM indomethacin) and stained with oil red-O. Scale bars, 50 µm. (*d*) Triglyceride (TG) accumulation was quantified and normalized to protein amount at day 8 of differentiation after transfection. (*e*) The expression of adipocyte genes (*PPARγ2, aP2, LPL* and *adiponectin*) at day 8 was detected by qPCR. (*f*) PPARγ and adiponectin protein levels at day 8 were detected by western blot. (*g*) The Bcl6 protein expression levels after transfection with pCDNA3.1-Bcl6 or control vector after 48 h. (*h*) Adipogenic phenotypes of C3H10T1/2 cells transiently transfected with pCDNA3.1-Bcl6 or control vector after being induced for 8 days with MDII, and stained with oil red-O. Scale bars, 50 µm. (*i*) Triglyceride accumulation was quantified and normalized to protein amount at day 8 of differentiation after transfection. (*j*) The expression of adipocyte genes (*PPARγ2, aP2* and *adiponectin*) at day 8 was detected by qPCR. Values are mean ± s.d. (*n* = 3). *p < 0.05, **p < 0.01 versus pCDNA3.1. (*k*) Adipogenic phenotypes of C3H10T1/2 cells transiently transfected with pRNAT-shBcl6 or pRNAT-shNC vector, or cotransfected with pRNAT-shBcl6 and pCMV-PPARγ, and stained with oil red-O at day 8 of adipogenic differentiation, Scale bars, 50 µm. (*l*) Triglyceride accumulation was quantified and normalized to protein amount at day 8; values are represented as mean ± s.d. (*n* = 3). *p < 0.05, **p < 0.01.

As the expression was markedly upregulated in the early stage of C3H10T1/2 cell adipogenesis, we speculated that Bcl6 would be a positive regulator of early adipose commitment. To determine whether Bcl6 is required for MSC adipogenic commitment and differentiation, C3H10T1/2 cells were transfected with siRNAs targeting *Bcl6* or NC siRNA. Efficacy of the RNAi in knockdown of *Bcl6* in C3H10T1/2 cells was confirmed by qPCR assay, the result showing both siRNAs have more than 50% reduction in mRNA transcripts by 36 h post-transfection (figure 2*a*). Furthermore, western blot analysis confirmed that Bcl6 protein level was decreased at 48 h post-transfection (figure 2*b*).

At day 8 post-induction, knockdown of *Bcl6* by both siRNA-1 and siRNA-2 blocked lipid accumulation and impaired mature adipocytic phenotype (figure 2*c*), and decreased the triglyceride contents (figure 2*d*). Consistent with the adipocytic phenotype, expression of the *PPARγ2* and adipocyte markers *aP2*, *LPL* and *adiponectin*, as well as protein expression of PPARγ and adiponectin were significantly downregulated in cells transfected with the siBcl6 compared with that in the cells transfected with siNC (figure 2*e,f*). We also verified the cell culture findings described above in a more physiological context [28,29]. Subconfluent WT or Bcl6 KD C3H10T1/2 stable cells were first treated with MDII and then implanted into athymic mice; results are illustrated in the electronic supplementary material, figure S1. After six weeks, the implanted WT C3H10T1/2 cells developed into tissue indistinguishable from epididymal adipose tissue of the same animal. Relatively, Bcl6 KD almost halved the formation of mature fat cells in the transplanted pad compared with the WT group and some small fat droplets appeared (electronic supplementary material, figure S1*a*). Moreover, the mRNA and protein expression of PPARγ and adipocyte markers aP2 and adiponectin from implants of injected Bcl6 KD C3H10T1/2 cells were significantly lower than those of WT cells (electronic supplementary material, figure S1*b*,*c*). These findings thus verify that Bcl6 is also highly important for adipogenesis *ex vivo*. Together, these results indicate that silencing of *Bcl6* is sufficient to decrease the rate of MSCs committed and differentiated into adipocytes.

Furthermore, we investigated the effect of *Bcl6* overexpression in C3H10T1/2 cells and observed a slight enhancement of the adipogenic phenotype when compared with the control cells after MDII induction (figure 2*h*). Moreover, the triglyceride contents and mRNA expression of *PPARγ2* and *aP2* in C3H10T1/2 cells with *Bcl6* overexpression was increased compared with the control cells (figure 2*i*,*j*). These results suggest that overexpression of Bcl6 promotes adipogenic potential of MSCs. Thus, the combined data from gain- and loss-of-function studies consistently demonstrate that Bcl6 acts as a promoter of adipogenesis.

We next sought to determine whether Bcl6 acts at an early or a late stage in the differentiation process. As shown previously, knockdown of *Bcl6* sharply repressed lipid accumulation and triglyceride contents in C3H10T1/2 cells, while this effect was largely reversed by coexpressing PPARγ (figure 2*k*,*l*). These data suggest that Bcl6 acts prior to PPARγ in the adipogenic differentiation cascade.

### 3.3. The ZF and PEST domain are involved in Bcl6 regulating adipogenesis

**Figure 3.**
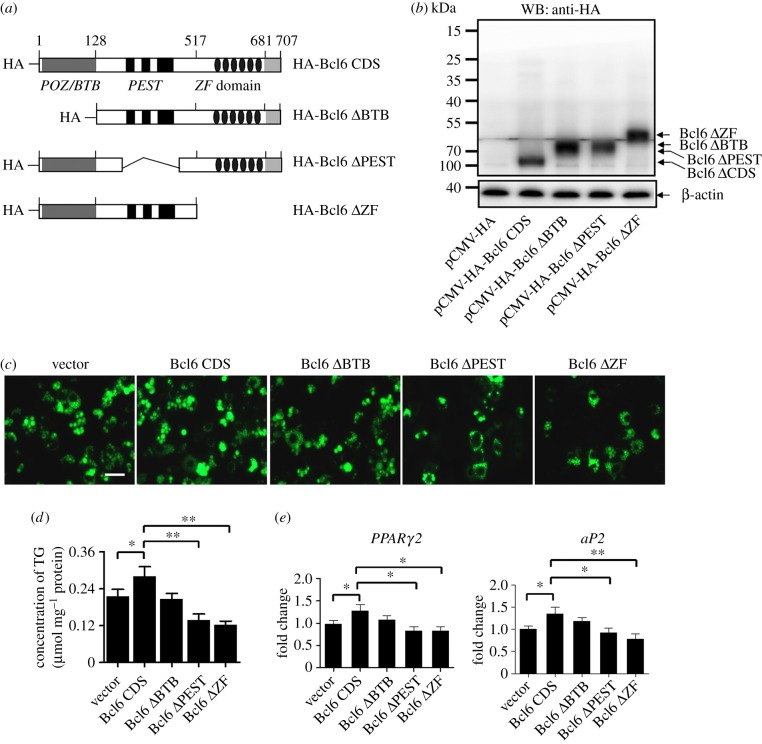
The ZF and PEST domain are required in Bcl6 regulating adipogenesis. (*a*) Schematic representation of Bcl6 domain truncation. (*b*) Bcl6 domain truncation plasmids were transfected into C3H10T1/2 cells, and the protein expression was detected by western blot after 48 h. (*c*) Adipogenic phenotypes of C3H10T1/2 cells transiently transfected with Bcl6 domain truncation plasmids or empty vector after being induced for 8 days with MDII. Lipid accumulation at day 8 was assessed by BODIPY 493/503 staining (green); the images were taken with a Leica DFC 300 (Germany) fluorescence microscope. Scale bar, 50 µm. (*d*) Triglyceride (TG) accumulation was quantified and normalized to protein amount at day 8 of differentiation. (*e*) The expression of adipocyte genes (*PPARγ2* and *aP2*) at day 8 was detected by qPCR. Values are mean ± s.d. (*n* = 3). *p < 0.05, **p < 0.01.

Bcl6 belongs to the ZF transcription factors, which have an amino-terminal POZ/BTB domain, a central PEST domain and six C2H2 ZF domains at the carboxy-terminus [30]. A large number of previous studies have indicated that Bcl6 mainly acts as a repressor of transcription involved in lymphocyte activation, differentiation, proliferation and migration [31–33]. To address whether Bcl6 acts as a transcriptional regulator and to uncover the novel aspects of biochemical mechanisms of Bcl6 in regulating adipogenesis, we generated Bcl6 domain-deleted plasmids to evaluate the contributions of different domains to the regulation of adipogenesis (figure 3*a*). The levels of expression of Bcl6 protein and its derivatives at 48 h post-transfection were confirmed by western blot analysis (figure 3*b*).

Next, Bcl6 and the derivative plasmids were transfected into C3H10T1/2 cells, and lipid accumulation was assessed by fluorescent dye BODIPY 493/503 staining at day 8 of adipogenic differentiation. Consistent with the result in figure 2*h*, transfected full-length Bcl6 vector slight increased the number of positive BODIPY 493/503-stained cells and the triglyceride contents (figure 3*c*,*d*). However, the number of adipogenic-differentiated cells was decreased after transfection with the vector of Bcl6 with PEST or ZF domain deleted, while there was no effect with loss of the POZ/BTB domain (figure 3*c*,*d*). Moreover, expression of *PPARγ2* and *aP2* was significantly downregulated in cells with the absence of the Bcl6 PEST or, in particular, the ZF domain, compared with that in the cells transfected with full-length Bcl6 (figure 3*e*). Taken together, these results suggest that the Bcl6 ZF and PEST domains serve as the primary components in regulating adipogenesis, while the POZ/BTB domain may make a minor contribution.

### 3.4. Illumina HiSeq 2500 sequencing and Bcl6 downstream targets

The Bcl6 ZF domain binds to DNA in a sequence-specific manner, and a consensus has been identified with core sequence TTCCT(A/C)GAA [17]. We proposed that Bcl6 acts as a transcriptional regulator so we tried to identify downstream targets of Bcl6 in adipogenesis of stem cells. Thus, we first assessed whether the expression of key adipogenic transcriptional regulators is sensitive to Bcl6 levels. Indeed, knockdown of *Bcl6* significantly downregulated the expression of *Zfp423*, *Zfp467*, *KLF15*, *PPARγ2* and *C/EBPα* in C3H10T1/2 cells (electronic supplementary material, figure S2*a*). Conversely, *Bcl6* overexpression increased *Zfp467*, *KLF15* and *C/EBPδ* levels in C3H10T1/2 cells (electronic supplementary material, figure S2*b*,*c*). These results suggest that Bcl6 may be a key transcriptional component of adipogenic regulatory networks.

**Figure 4.**
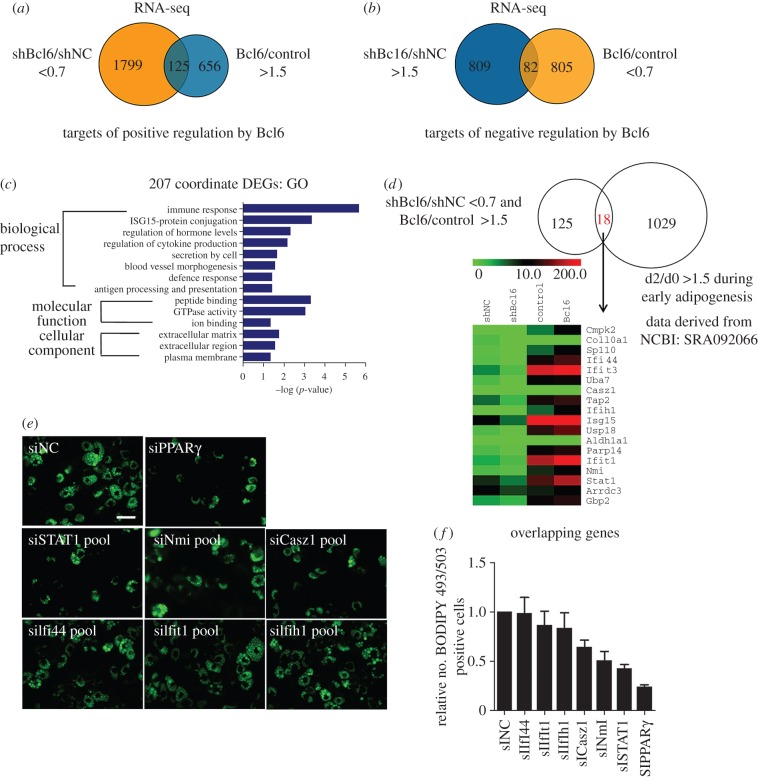
Identifying downstream targets of Bcl6 in C3H10T1/2 cells by RNA-seq. C3H10T1/2 cells were transduced with vectors expressing shBCL6, shNC, pCDNA3.1-Bcl6 or empty vector. After G418 selection, total cellular RNA was extracted and submitted for analysis using RNA-seq. (*a*,*b*) The Venn diagram shows the number of genes positively regulated by shBcl6 (shBcl6/shNC less than 0.7-fold and Bcl6/control more than 1.5-fold) and negatively regulated by Bcl6 (shBcl6/shNC more than 1.5-fold and Bcl6/Control less than 0.7-fold), respectively. (*c*) GO functional enrichment analysis of 207 co-ordinate DEGs. The results are summarized in the following three main categories: biological process, molecular function and cellular component. The *y*-axis indicates functional groups. The *x*-axis indicates –log(*p-*value). (*d*) The Venn diagram shows the 18 overlapping DEGs that are positively regulated by Bcl6 (i.e. shBcl6/shNC less than 0.7-fold and Bcl6/control more than 1.5-fold) and upregulated during early adipogenesis. The heat map corresponds to genes in the intersecting set. (*e*) Effect of Bcl6 targets knockdown by pooled siRNAs on adipogenic phenotypes of C3H10T1/2 cells. Lipid accumulation at day 8 was assessed by BODIPY 493/503 staining and the images were taken with a Leica DFC 300 (Germany). Scale bar, 50 µm. (*f*) The number of the BODIPY 493/503 positive cells in the photograph was counted by CellProfiler Image (NIH, USA); the ratio of the treatment and control groups are represented as mean ± s.d. *n* = 10 photographs were taken under different view.

To gain global insights into gene expression alterations dependent on Bcl6 levels, we performed RNA-seq experiments in both gain- and loss-of-function contexts to enhance our ability to identify bona fide targets of Bcl6, focusing on differential expression genes (DEGs) that showed coordinate regulation between *Bcl6* overexpression and knockdown. Validation of RNA-Seq-based gene expression by qPCR suggested that the results of RNA-Seq analysis were reliable indicators of overall changes in gene expression (electronic supplementary material, figure S3). A total of 1799 genes had their expression fall below 70% of baseline when *Bcl6* was targeted by RNAi, whereas the expression of 656 genes was increased by more than 1.5-fold when *Bcl6* was overexpressed (figure 4*a*). A total of 125 genes showed coordinate regulation, thereby suggesting that these are *bona fide* positive targets of Bcl6. Conversely, 809 genes showed increased expression upon treatment with shBcl6, while 805 genes had diminished expression with *Bcl6* overexpression (figure 4*b*); 82 of these genes were coordinately regulated. We next preformed GO analysis of 207 coordinate DEGs to further understand the biological functions of the targets of Bcl6 in C3H10T1/2 cells. Results showed that these genes were clustered into 14 significant GO categories, which were predominantly involved in immune response, ISG15-protein conjugation, regulation of hormone levels, regulation of cytokine production and binding processes (figure 4*c*).

In order to better identify which genes are required for the adipogenic transcriptional programme and *bona fide* direct targets of Bcl6 promotion, we mapped the 207 coordinate DEGs (electronic supplementary material, dataset S1) to the upregulated genes during early adipogenesis (NCBI GEO datasets: SRA092066), thus obtaining 18 overlapping genes that might be targets of Bcl6 during adipogenesis (figure 4*d*). Furthermore, to establish the direct transcriptional effects of Bcl6 on these overlapping genes, we identified six key factors which have potential Bcl6 binding sites on their promoter through bioinformatics analysis (electronic supplementary material, table S5). Among these six targets, *STAT1* was of particular interest to us, since RNAi-mediated knockdown of *STAT1* expression significantly inhibited adipogenic phenotype more obviously than knockdown of others genes in C3H10T1/2 cells, an effect that mimicked that of PPARγ knockdown (figure 4*e*,*f*).

### 3.5. Bcl6 transactivates *STAT1* by directly binding to the *STAT1* promoter

**Figure 5.**
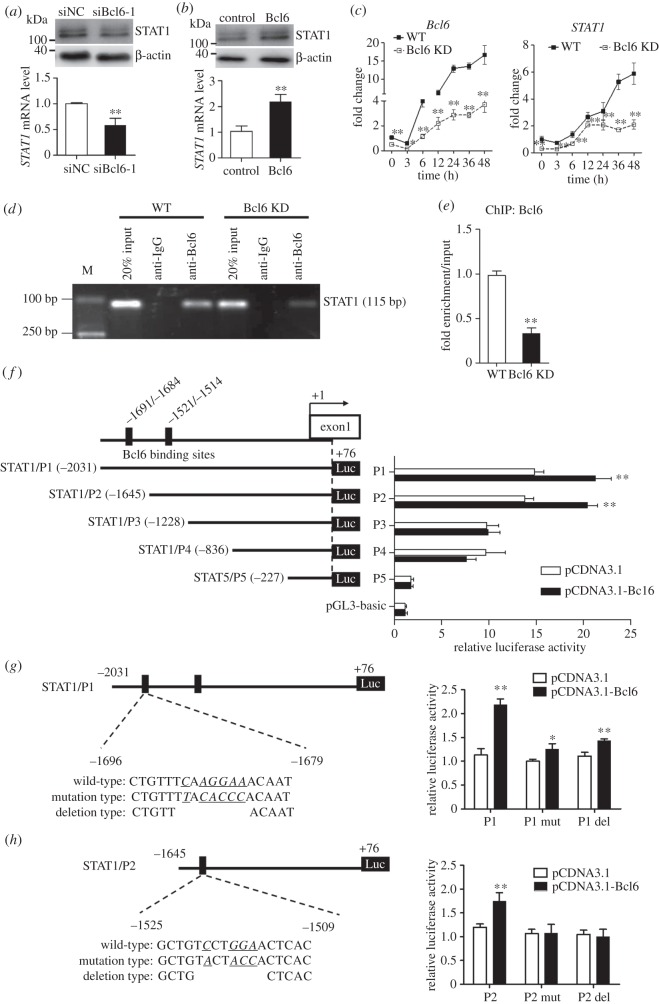
*STAT1* is a direct target of Bcl6 in C3H10T1/2 cells. (*a*,*b*) Knockdown of Bcl6 in C3H10T1/2 cells represses STAT1 expression, and overexpression of Bcl6 enhances STAT1 expression by qPCR and western blot, respectively. Values are mean ± s.d. (*n* = 3). **p < 0.01. (*c*) Subconfluent WT and Bcl6 KD C3H10T1/2 cells were treated with MDII, and then cells were collected at 0, 3, 6, 12, 24, 36 and 48 h, respectively. The mRNA expression of *Bcl6* and *STAT1* was detected by qPCR at various time points. Values are mean ± s.d. (*n* = 3). *p < 0.05, **p < 0.01 versus WT. (*d*) ChIP assay of Bcl6 binding to *STAT1* promoter. WT and Bcl6 KD C3H10T1/2 cells were treated with MDII for 48 h, and then ChIP assays were performed. Total chromatins were indicated as input, pre-immune IgG was used as an NC. (*e*) After immunoprecipitation, potential region on *STAT1* promoter for Bcl6 binding in WT or Bcl6 KD cells was amplified by qPCR. Twenty per cent input was internal reference. Values are mean ± s.d. (*n* = 3). **p < 0.01. (*f*) The truncated *STAT1* promoter reporters (P1∼P5) were co-transfected with pCDNA3.1-Bcl6 or empty vector (pCDNA3.1) into C3H10T1/2 cells. The luciferase reporter activity was measured 48 h after transfection. (*g,h*) Schematics structure of the first and second Bcl6 binding site mutation and deletion, respectively. The histogram represents effect of Bcl6 binding site mutation and deletion on P1 and P2 promoter activity. Wild-type (P1 or P2), mutation type (P1 or P2 mut) and deletion type (P1 or P2 del) of *STAT1* promoter reporters were, respectively, co-transfected with pcDNA3.1-Bcl6 into C3H10T1/2 cells. The dual-luciferase activity was measured 36 h after transfection. Values are represented as mean ± s.d. (*n* = 6). *p < 0.05, **p < 0.01 versus pCDNA3.1.

As shown in figure 5*a*,*b*, qPCR and western blot analysis in C3H10T1/2 cells confirmed that Bcl6 exerted strong positive effects on *STAT1* expression, whereas reducing the *Bcl6* expression had the converse effect. Moreover, during the early adipogenesis of WT or Bcl6 KD C3H10T1/2 cells, the mRNA expression pattern of *STAT1* was coordinated with *Bcl6*. Both were upregulated in the early stage and peaked at 48 h, whereas the knockdown of *Bcl6* significantly inhibited S*TAT1* expression (figure 5*c*).

To assess whether Bcl6 specifically binds to the *STAT1* promoter during early adipogenesis, we analysed the binding activity of Bcl6 to the endogenous *STAT1* promoter in C3H10T1/2 stem cells by ChIP assays. ChIP results showed significant Bcl6 binding to the *STAT1* promoter, which was detected at day 2 after MDII induction, whereas knockdown of *Bcl6* significantly decreased the fragment enrichment in Bcl6 KD C3H10T1/2 cells (figure 5*d*,*e*).

We next examined whether Bcl6 directly regulates the transcription of the *STAT1* gene, and identified the region responsible for Bcl6 transactivation by promoter activity experiments. A series of deletions (P1, −2031/+76 bp; P2, −1645/+76 bp; P3, −1228/+76 bp; P4, −836/+76 bp; P5, −227/+76 bp) of a 2107 bp fragment of mouse *STAT1* promoter were constructed and cotransfected into C3H10T1/2 cells with *Bcl6* overexpression plasmid, respectively (figure 5*f*). Among the five promoters, only P1 and P2 promoters contained the potential binding site for Bcl6. Figure 5*f* shows that Bcl6 significantly enhanced P1 and P2 promoter activity in reporter assays, whereas the activation was almost completely abolished when the P3, P4 and P5 promoters were used. These results indicate that Bcl6 plays a critical role in mouse *STAT1* promoter activity and the direct binding site exists between −2031 and −1228 bp in the mouse *STAT1* promoter.

According to the bioinformatics analysis, the *STAT1* promoter in mouse contains two potential binding sites for Bcl6 in the −2031 and −1228 bp region, whose core sequences were TCAAGGAA and TCCTGGAA, respectively. Site-specific mutation and deletion in both P1 and P2 promoters were performed to analyse the activity of mouse *STAT1* promoters by the luciferase reporter assay in C3H10T1/2 cells. The results showed that either mutation or deletion attenuated the P1 promoter activity, whereas mutation or deletion in the P2 promoter completely reduced the luciferase activity (figure 5*g*,*h*). These results suggest that these two potential binding sequences may be functionally important.

To verify the regulatory relationships between Bcl6 and *STAT1* in a more physiological context, we analysed their expression in the fat tissues of high-fat diet-induced obese mice. Results showed that the expression of *Bcl6* was significantly increased in white fat tissues but was decreased in brown fat; the expression pattern of *STAT1* coincided with that of *Bcl6* (electronic supplementary material, figure S4). This result suggests that *STAT1* may be a direct target gene of Bcl6 *in vivo* during adipogenesis.

### 3.6. STAT1 is required for adipogenesis of C3H10T1/2 cells

**Figure 6.**
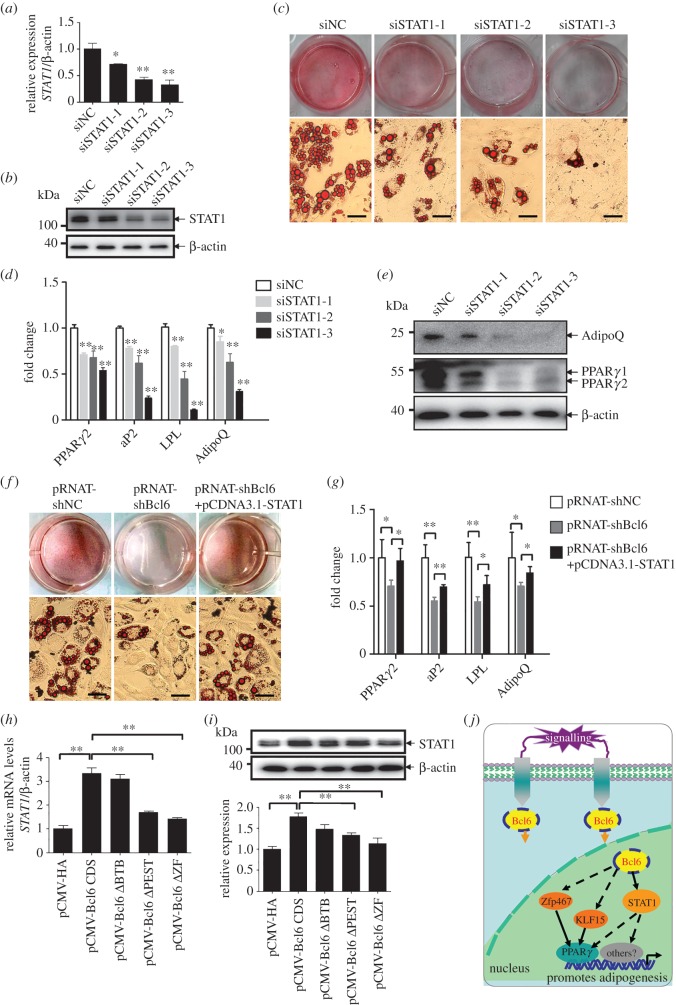
STAT1 is required during adipogenesis and rescues the adipogenic competency in Bcl6 knockdown C3H10T1/2 cells. (*a*,*b*) The *STAT1* mRNA and protein expression levels after transfected siNC or siSTAT1s after 36 h and 48 h, respectively. (*c*) Adipogenic phenotypes of C3H10T1/2 cells transiently transfected with synthetic siRNAs targeting *STAT1* or NC siRNA after induced 8 days by MDII and stained with oil red-O, Scale bars, 50 µm. (*d*) The expression of adipocyte genes *PPARγ2, aP2, LPL* and *adiponectin* at day 8 was determined by qPCR. (*e*) The expression of adipocyte protein PPARγ and adiponectin at day 8 was determined by western blot. (*f*) Adipogenic phenotypes of C3H10T1/2 cells transiently transfected with pRNAT-shBcl6 or pRNAT-shNC, or cotransfected with pRNAT-shBcl6 and pCDNA3.1-STAT1 after induced by MDII, and stained with Oil red-O on day 8 of adipogenic differentiation, Scale bars, 50 µm. (*g*) The expression of adipocyte genes *PPARγ2, aP2, LPL* and *adiponectin* was detected by qPCR. (*h,i*) The mRNA and protein expression levels of STAT1 after transfected with Bcl6 domain truncation plasmids or empty vector after 36 h and 48 h, respectively. Values are represented as mean ± s.d. (*n* = 3). *p < 0.05, **p < 0.01. (*j*) A proposed model for the transcriptional cascade involving Bcl6 and STAT1 in adipogenesis. Bcl6 positive regulates early adipogenesis is at least partially mediated by the direct promotion of *STAT1* and indirect enhancement of *Zfp467* and *KLF15* expression. Zfp467 [10] and KLF15 [34] have been reported to direct regulate the *PPARγ* expression during adipogenesis. STAT1 may indirectly regulate the expression of *PPARγ* and other pro-adipogenic factors. Solid lines indicate directly regulation of gene expression, whereas dashed lines indicate indirectly regulation of gene expression.

Since *STAT1* was shown to be a target of Bcl6 during adipogenesis, its principal role was further evaluated by its knockdown in C3H10T1/2 cells. The efficacy of the RNAi to knockdown *STAT1* in C3H10T1/2 cells was confirmed by qPCR and western blot, respectively (figure 6*a*,*b*). At day 8 post-induction, oil red-O staining revealed the dramatic impairment in adipocytic phenotypes for individual siRNAs and almost completely abolished differentiation when siSTAT1-3 was used (figure 6*c*). Correspondingly, the expression of *PPARγ2* and adipocyte markers *aP2*, *LPL* and *adiponectin*, as well as protein expression of PPARγ and adiponectin, was significantly downregulated in cells transfected with the individual *STAT1* siRNA as compared with those transfected with NC siRNA (figure 6*d*,*e*). These results indicate that STAT1 is required for the adipogenesis of C3H10T1/2 cells.

Because *STAT1* was a direct target of Bcl6 and is required for adipogenesis of pluripotent mesenchymal cells, we further evaluated its role by overexpressing *STAT1* in *Bcl6* knockdown C3H10T1/2 cells. As expected, the reduced expression of *Bcl6* inhibited the adipocytic commitment and differentiation of C3H10T1/2 cells, which was observed by oil red-O staining (figure 6*f*); the expression of *PPARγ2*, *aP2*, *LPL* and *adiponectin* was also decreased after MDII induction (figure 6*g*). However, the decreased adipocyte gene expression (*PPARγ2*, *aP2*, *LPL* and *adiponectin*) as a consequence of *Bcl6* knockdown was reversed by the coexpression of *STAT1* (figure 6*f*,*g*). Therefore, the overexpression of *STAT1* could rescue the impairment of adipogenic commitment and differentiation induced by *Bcl6* knockdown in C3H10T1/2 cells.

In addition, we investigated the effect of the Bcl6 key domain truncation on the expression of the direct target *STAT1*. The results showed that the absence of the ZF or PEST domain blocked the mRNA and protein expression of STAT1 (figure 6*h*,*i*). Our observations thus demonstrate that Bcl6 plays an essential role in adipogenesis in C3H10T1/2 cells through its regulation of *STAT1* expression. The absence of the PEST or ZF domain of Bcl6 attenuates adipogenic commitment and differentiation, as well as the expression of *STAT1* in C3H10T1/2 cells.

## 4. Discussion

The increase of adipose tissue mass associated with obesity is due in part to an increase in adipocyte formation from MSCs [3,35]. Identifying the factors that regulate the adipogenesis should provide insight into the mechanisms by which pluripotent MSCs undergo commitment to the adipose lineage. In this study, we used the candidate gene approach to identify novel factors by focusing on the TFs involved in the regulation of early adipogenesis. We targeted the Zfp Bcl6, which acts as a potent positive regulator of early adipogenesis, owing to it being required in adipogenic phenotypes of porcine SV cells. Although a previous study reported that Bcl6 could regulate adipose tissue development by inhibiting the expression of suppressor of cytokine signalling (Socs) 2 during lipid metabolism [22], its roles in adipocyte lineage commitment and adipogenesis remain poorly understood.

This study first showed that *Bcl6* is enriched in preadipocytes and upregulated; in particular, it is expressed earlier than *PPARγ2* during the early stage of MSC adipogenesis (figure 1*a*,*c*,*d*), suggesting that Bcl6 may be a key factor during early adipogenesis. Then we directly determined the key role of Bcl6 on the regulation of adipogenic commitment and differentiation of MSCs *in vitro* and *ex vivo*. The gain- and loss-of-function studies highlighted the role of Bcl6 in early adipogenesis and characterized it as a novel positive transcriptional regulator (figure 2; electronic supplementary material, figure S1), which exerts its effect on regulating adipogenesis mainly dependent on the ZF and PEST domains (figure 3). Previous work has revealed that the Bcl6 PEST domain was associated with protein interactions that mediated the interaction of Bcl6 with multiple proteins, such as p300 [36], MTA-3 [37] and CtBP [38], to influence different biological events. This suggests that in MSC adipogenesis Bcl6 may interact with one or more pro-adipogenic factors via its PEST domain to promote adipogenic commitment and differentiation, though the Bcl6-interacting protein during adipogenesis has not been identified in this study.

Similar to other well-characterized early pro-adipogenic Zfps, such as Zfp423 [8], Zfp467 [10] and ZEB1 [14], Bcl6 contains six potent C2H2-ZF motifs which enable this protein to bind directly to specific DNA sequences [17,39]. Thus, we asked how Bcl6 acts as a TF to exercise the positive regulation during early adipogenesis. First, we addressed the transcriptional regulation of Bcl6 on the expression of well-characterized pro-adipogenic factors. The results showed that reducing Bcl6 significantly downregulates the expression of *Zfp423*, *Zfp467*, *KLF15*, *PPARγ2* and *C/EBPα*, whereas Bcl6 overexpression increases *Zfp467*, *KLF15* and *C/EBPδ* levels in C3H10T1/2 cells (electronic supplementary material, figure S2). These results suggest that Bcl6 may act upstream of these pro-adipogenic factors during the adipogenic differentiation cascade. Moreover, we speculated that *Zfp467* and *KLF15* may be direct targets of Bcl6 during early adipogenesis, since they are concordantly regulated by knockdown and overexpression of *Bcl6*. Nevertheless, the ChIP experiments confirmed that the binding of Bcl6 to the *Zfp467* or *KLF15* promoter is not detected by the Bcl6-specific antibody in C3H10T1/2 stem cells, suggesting that they may be indirect targets of Bcl6 during adipogenesis (X Hu, Y Yang 2015, unpublished data).

To further identify *bona fide* direct targets of Bcl6, the second strategy was to use large-scale gene expression screening methods, such as RNA-seq, to find genes whose expression is altered by the presence or the absence of Bcl6, and then verify whether there is a Bcl6 binding site in the promoter of the gene. The data strongly implied a mechanism involving the transcriptional activation of the new factor STAT1, although other targets are certainly possible. STAT1 is a member of the STAT family that mediates a variety of physiological processes, including development, haematopoiesis, cell death and inflammatory responses [40–42]. In regulation of fat biological processes, Stephens *et al.* [43] found that protein levels of STAT1, -3 and -5 increased during 3T3-L1 fat cell differentiation, and Stewart *et al.* [44] reported that the expression of STAT1, -5A and -5B tightly correlates with lipid accumulation and the expression of both C/EBPα and PPARγ during adipogenesis. More recent studies showed that STAT3 functions as a critical factor for 3T3-L1 adipogenesis via a mechanism involving the PPARγ activation pathway [45], and STAT5A expression in Swiss 3T3 cells promotes adipogenesis *ex vivo* in an athymic mice model system [46]. However, there is no available study to date that examines the role of STAT1 in the transcriptional control of early adipogenesis.

Our results showed that the mRNA expression of STAT1 increased during the early stage of adipogenesis (figure 5*c*), and RNAi-mediated knockdown of *STAT1* inhibited adipogenic commitment and differentiation of C3H10T1/2 cells (figure 6*a–e*), confirming that STAT1 is the key factor of early adipogenesis. Further research revealed that STAT1 expression patterns were consistent with those of Bcl6 both in the early adipogenesis of C3H10T1/2 cells and the fat development of high-fat diet-induced obese mice; the knockdown of *Bcl6* also significantly inhibits the S*TAT1* expression in undifferentiated or differentiated C3H10T1/2 cells (figure 5*a*–*c*; electronic supplementary material, figure S4). However, this result was inconsistent with a previous report that S*TAT1* expression is inhibited by Bcl6 in osteoblasts [20]. Bcl6 may positively regulate *STAT1* expression via the Bcl6-interacting protein in MSCs, but this effect is dependent on the cell type and cell context.

According to the ChIP experiments and promoter activity assay, we confirmed that Bcl6 can transactivate *STAT1* by directly binding to the −2031 and −1228 bp regions of the mouse *STAT1* promoter (figure 5*d*–*h*). Therefore, *STAT1* is probably a direct downstream target of Bcl6 during the early adipogenesis of stem cells. Furthermore, results indicated that *STAT1* overexpression reversed defects in adipogenesis caused by *Bcl6* knockdown. In addition, based on the promoter binding prediction, the promoters of well-known adipogenic genes (such as *Zfp423*, *Zfp467*, *Ebf1*, *KLF15* and *PPARγ*) have one or more potential STAT1 binding sites (electronic supplementary material, table S6). Stewart *et al.* [44] also reported that the expression of STAT1 is regulated in an identical manner to both PPARγ and C/EBPα by TNFα and thiazolidinedione, suggesting that STAT1 may play a role in the regulation of adipocyte gene expression [44]. Hence, STAT1 may directly or indirectly regulate the expression of pro-adipogenic factors in adipogenesis.

In conclusion, our study demonstrated an essential role of Bcl6 in the complex regulation of gene transcription during early adipogenesis. The action of Bcl6 is at least partially mediated by the direct promotion of *STAT1* and indirect enhancement of *KLF15 and Zfp467* expression (figure 6*j*).

Adipocytes originate from MSCs, which are also precursors for muscle, cartilage and bone cells [47]. Zfps have emerged as a set of key transcriptional regulators during early adipogenic commitment of MSCs, and an increasing number of Zfps have been associated with early adipogenesis [48]. Our findings report a novel Zfp related to early adipogenesis and identify Bcl6 as a key pro-adipogenic TF. The elucidation of the mechanisms that control Bcl6 expression could further clarify the underlying mechanisms of adipogenesis, thereby providing novel insights to prevent the growing incidence of obesity and metabolic syndromes in the modern world.

## Supplementary Material

Identification of zinc finger protein Bcl6 as a novel regulator of early adipose commitment

## Supplementary Material

Supplementary material_dataset S1
