# Supplementary material for: Identification of zinc finger protein Bcl6 as a novel regulator of early adipose commitment
Source: Open Biol. 2016 Jun 1;6(6):160065. doi: 10.1098/rsob.160065 (PMC4929941; doi:10.1098/rsob.160065)
Supplement: Identification of zinc finger protein Bcl6 as a novel regulator of early adipose commitment [file rsob160065supp1.pdf]

Supplementary material

Identification of zinc finger protein Bcl6 as a novel regulator of early adipose commitment

Xiaoming Hu [1,3], Yuanfei Zhou [1], Yang Yang [1], Jie Peng [1], Tongxing Song [1], Tao Xu [1], Hongkui, Wei [1], Jian Peng [1,3,*], Siwen Jiang [2,3]

[1]Department of Animal Nutrition and Feed Science, College of Animal Science and Technology, Huazhong Agricultural University, Wuhan 430070, People's Republic of China

[2]Key Laboratory of Animal Genetics, Breeding and Reproduction of Ministry of Education, College of Animal Science and Technology, Huazhong Agricultural University, Wuhan, 430070, People's Republic of China

[3]The Cooperative Innovation Center for Sustainable Pig Production, Wuhan 430070, People's Republic of China

[*]Corresponding authors at: Department of Animal Nutrition and Feed Science, College of Animal Science and Technology, Huazhong Agricultural University, Wuhan 430070, People's Republic of China. Tel.: +86 27 87280122, fax: +86 27 87281378 (J. Peng). E-mail: pengjian@mail.hzau.edu.cn (J.P.).

Supplementary material Materials and Methods

Mice

The tissue samples for gene expression assay obtained from 1-week-old and 8-week-old mice (129 /ICR background). Eight-week-old Kunming mice were fed either a control diet (D12450B; 20% protein, 70% carbohydrate, and 10% fat) or a high fat diet (HFD) (D12451; 20% protein, 35% carbohydrate, and 45% fat) for 8 weeks (Research Diets Inc.). All the experimental procedures were approved by the Hubei Committee on Laboratory Animal Care.

Hematoxylin and eosin staining

Epididymal white adipose tissue (epi-WAT) and brown adipose tissue (BAT) were fixed with 10% neutralbuffered formalin, embedded in paraffin, and sectioned. Hematoxylin and eosin (H&E) staining was then performed on these sections.

Stem cell s.c. implantation, excision of fat pads, and histology

The use and care of animals were approved by the Institutional Animal Care and Use Committee. To induce commitment, post-confluent Bcl6 knockdown (Bcl6 KD) C3H10T1/2 and wild type (WT) C3H10T1/2 cells were provided with DMEM containing 10% FBS, 1 μM dexamethasone, 0.5 mM 3–isobutyl-1–methylxanthine, 10 μg/ml insulin, and 200 μM indomethacin for 2 days, then trypsinized and collected. After centrifugation, cell pellets were resuspended in 10% FBS and injected s.c. (3×10^7 cells per site) with a 17-gauge needle at the sternum of Crl:NU/NU-nuBR athymic mice (five animals per group,

10-week-old, male; Charles River Breeding Laboratories). Mice were housed in microisolator cages. At 6 wk the mice were killed by cervical dislocation, and the fat pads derived from the implanted cells and epididymal fat pads were excised and fixed in neutral-buffered formalin. For light microscopy, fat pads derived from implanted stem cells and epididymal fat pads were paraffin-embedded after 24 h of fixation in buffered formalin. Paraffin tissue sections (4 μm) were stained with hematoxylin and eosin for histological analysis.

Statistical analysis

Values are expressed as means ± SD of at least three independent experiments. Prism was used to evaluate the data for statistical significance by two-tailed Student’s t-tests with $*P < 0.05$ (versus the indicated controls) considered as significant.

Supplementary material Figures

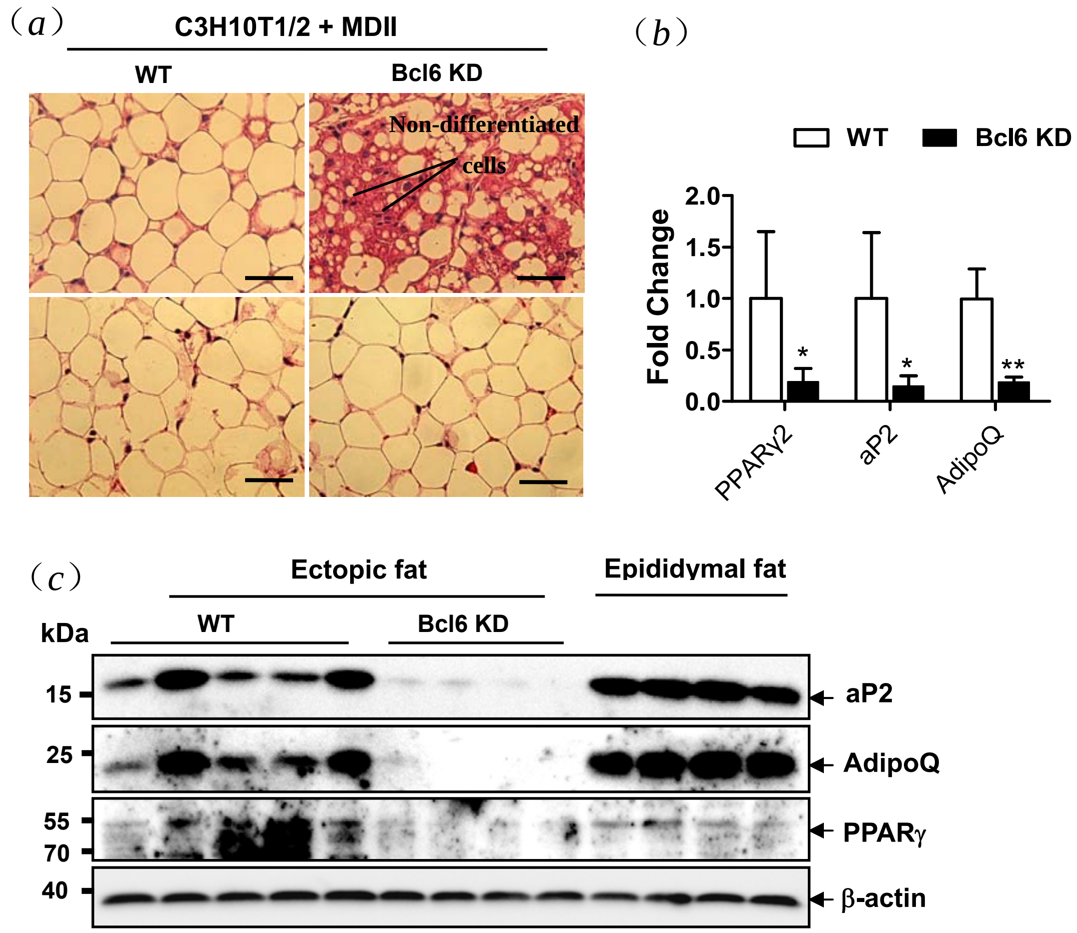

Figure S1. Reduction of Bcl6 inhibits adipogenic potential *ex vivo*. Subconfluent WT and Bcl6-KD C3H10T1/2 stem cells were treated with MDII for 2 days, trypsinized, and resuspended in 10% FBS and injected s.c. (3×10^7 cells per site) with a 17-gauge needle at the sternum of 10-week-old male athymic mice (Crl:NU/NU-nuBR). (*a*) Fat pads derived from the implanted cells (upper panels) and epididymal fat pads (positive control; lower panels) were excised and fixed in neutral-buffered formalin. Hematoxylin (blue) and Eosin (pink)-stained fat sections from representative samples of mouse Bcl6 knockdown transplants as well as the corresponding control (WT). Scale bar, 50 μm. (*b*) Ectopic fat pads found at the site of injection of WT (n=5) or Bcl6-KD (n=4) C3H10T1/2 cells were rapidly excised from athymic mice used for RNA and protein extracting. The relative expression of adipocyte genes *PPARγ2, aP2* and *AdipoQ* were determined by qPCR. The values are represented as means ± SD. *$P < 0.05$; **$P < 0.01$. (*c*) Adipocyte markers aP2, AdipoQ and PPARγ protein were determined by Western blot.

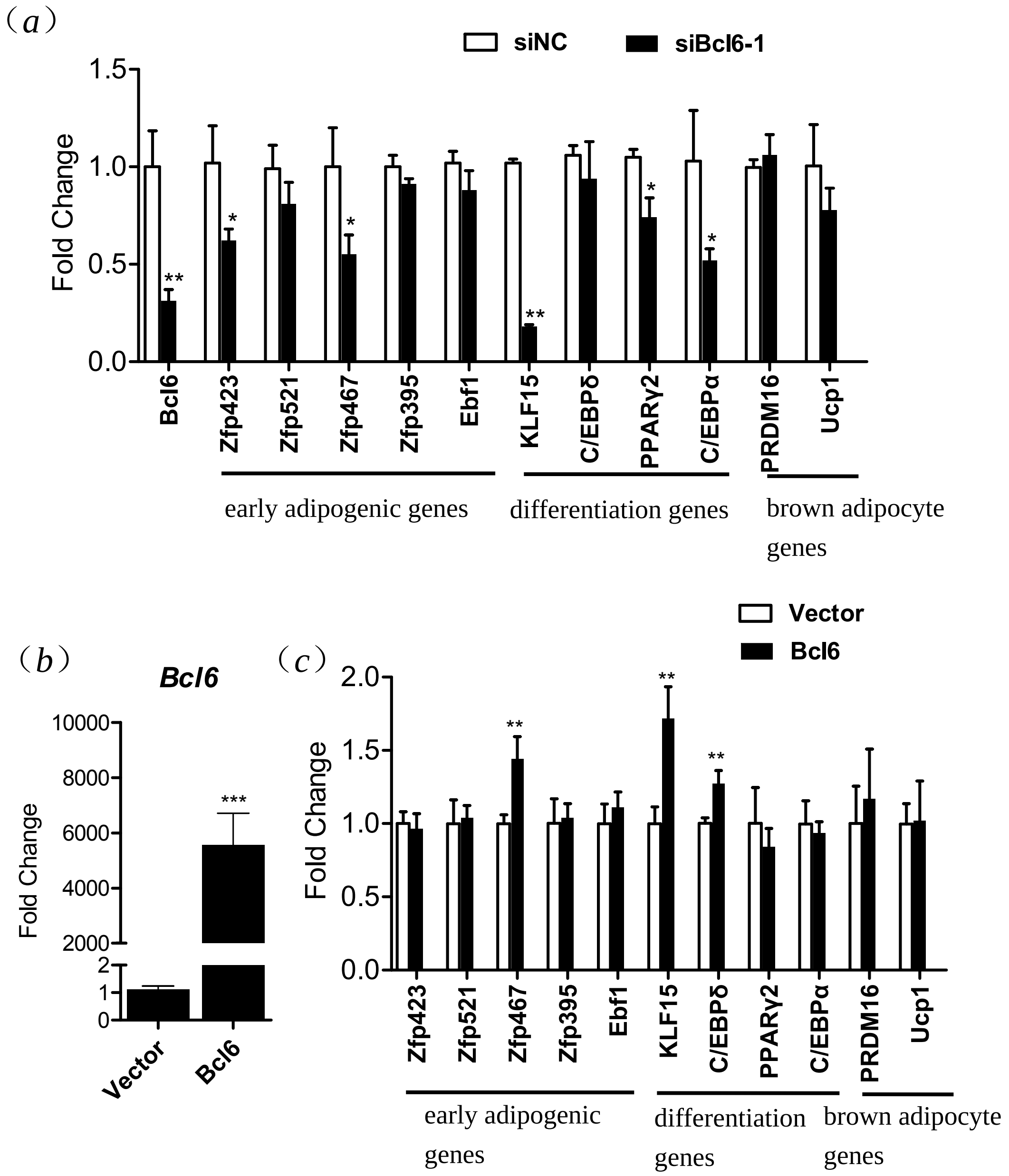

Figure S2. The effect of knockdown or overexpression of *Bcl6* on the mRNA expression of adipogenic genes. (*a*) The mRNA expression levels of adipogenic genes after transfected siRNA 36h. (*b*) The *Bcl6* expression after transfected pCDNA3.1-Bcl6 48h. (*c*) The mRNA expression levels of adipogenic genes after transfected pCDNA3.1-Bcl6 48h. Values are represented as means ± SD (n=3). **P* < 0.05, ***P* < 0.01, ****P* < 0.001 vs. siNC or empty vector.

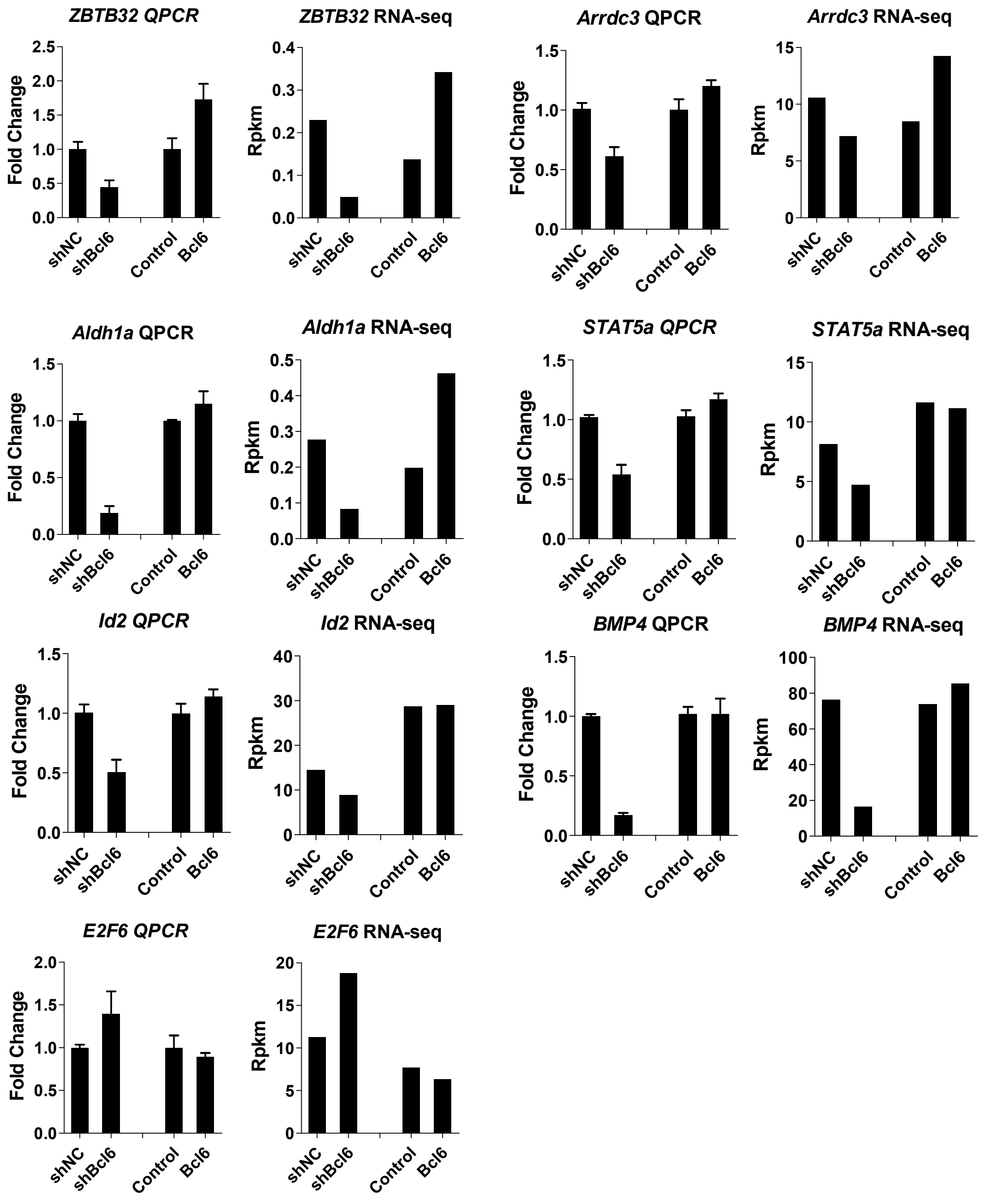

Figure S3. Validation of RNA-Seq-based gene expression. QPCR and RNA-Seq results of the expression mode of seven genes (*ZBTB32*、*Arrdc3*、*Aldh1a*、*STAT5a*、*Id2*、*BMP4* and *E2F6*) during knockdown of *Bcl6* with shRNA and overexpression of *Bcl6*. The values of relative expression levels are represented as means ± SD (n=3).

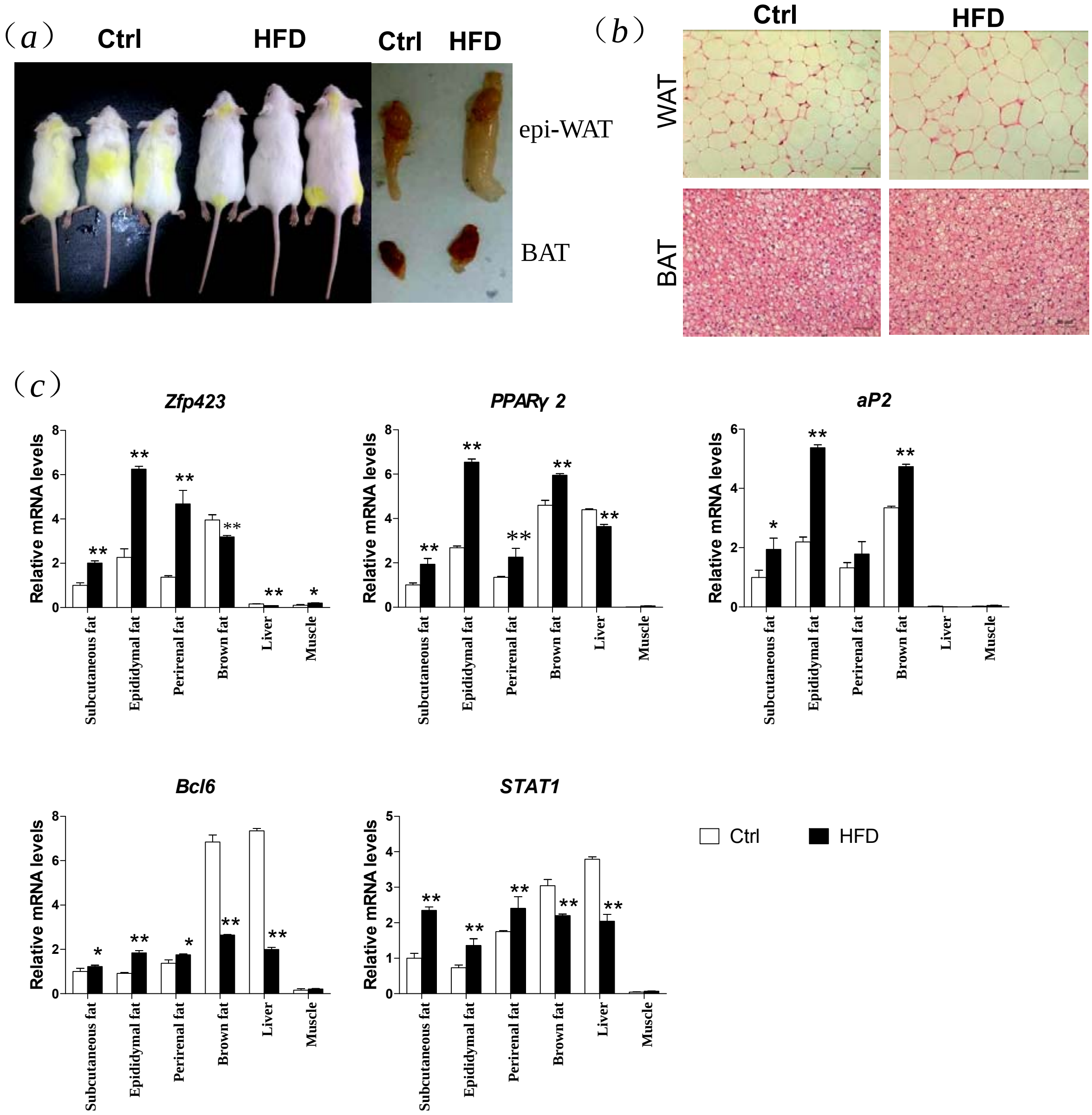

Figure S4. *STAT1* and *Bcl6* expression was coordinate upregulated in the white adipose tissue of HFD-induced obesity mice. After feeded 8 weeks by normal (Ctrl) or high-fat diet (HFD), mice were killed for collecting the subcutaneous, epididymal, perirenal and brown adipose tissue, and liver, muscle (n=5). (*a*) the mouse body type and fat mass of epididymal and brown fat tissue after 8 wk. (*b*) The development status of epididymal and brown fat tissue in Ctrl and HFD mice by H&E staining. Scale bar, 50 μm. (*c*) The mRNA expression of adipocyte genes *Zfp423*, *PPARγ2*, *aP2*, *Bcl6*, and *STAT1* was detected by qPCR at various tissues. Values are represented as means ± SD (n=5). *P < 0.05; **P < 0.01 vs. Ctrl.

Supplementary material Tables

Table S1 PCR primers for constructing overexpression vector

**Overexpression plasmids**	**PCR primers 5'-3'**
pCDNA3.1-Bcl6	Fw:*ccc*AAGCTTGCCATGGCCTCCCCGGCTGACAG<br>Rev:*cg*GGATCCTCAGCAGGCTTTGGGGAGCTCCG
pCDNA3.1-STAT1	Fw:*ccc*AAGCTTGCCATGTCACAGTGGTTCGAGCTTC<br>Rev:*cg*GGATCCTTATACTGTGCTCATCATACTGTC
pCMV-HA-Bcl6 CDS	Fw:*ga*AGATCTTC GCCTCCCCGGCTGACAG<br>Rev:*gg*GGTACCTCAGCAGGCTTTGGGAAGCTCCG
pCMV-HA-Bcl6 ΔBTB	Fw:*ga*AGATCTTCGAAGCAGAAATGGCCCCTGCACTTAAACC<br>Rev:*gg*GGTACCTCAGCAGGCTTTGGGAAGCTCCG
pCMV-HA-Bcl6 ΔPEST	Part1 Fw:*ga*AGATCTTCGCCTCCCCGGCTGACAG<br>Rev:TGCTGCTTCGCTGGGGACTAACCAGACCCTTCCG<br>Part2 Fw:TTAGTCCCCAGCGAAGCAGCAGTGAGAGTCACTCAC<br>Rev:*gg*GGTACCTCAGCAGGCTTTGGGAAGCTCCG
pCMV-HA-Bcl6 ΔZF	Fw:*ga*AGATCTTC GCCTCCCCGGCTGACAG<br>Rev:*gg*GGTACCTCAGGTCCCATTCTCACAGCTAGAATCCG

fw: forward primer, rev: reverse primer.

Table S2 PCR primers for promoter reporter vector

**Plasmid**	**Location**	**PCR primers 5'-3'**
STAT1/P1	-2031 ~ +76	Fw:*gg*GGTACCAGAAAGTGATTTCCTTCCAGAAAC<br>Rev:*cta*GCTAGCCAGGGAGCTTTGACAGACTC
STAT1/P2	-1645 ~ +76	Fw:*gg*GGTACCGTTGGTCCTCTGTGATATCTATG<br>Rev:*cta*GCTAGCCAGGGAGCTTTGACAGACTC
STAT1/P3	-1228~ +76	Fw:*gg*GGTACCAAGAAAGTGATAGGGAACATGG<br>Rev:*cta*GCTAGCCAGGGAGCTTTGACAGACTC
STAT1/P4	-836 ~ +76	Fw:*gg*GGTACCACAGACTCAGTAGATATCCAAGG<br>Rev:*cta*GCTAGCCAGGGAGCTTTGACAGACTC
STAT1/P5	-227 ~ +76	Fw:*gg*GGTACCGTACGTGAAGGTTAATCTCTAG<br>Rev:*cta*GCTAGCCAGGGAGCTTTGACAGACTC
STAT1/P1 mut		Fw:CCATCTGTTTTACACCCACAATTGATTCTC<br>Rev:TGTGGGTGTAAAACAGATGGAATTTGAATC
STAT1/P1 del		Fw:CCATCTGTTACAATTGATTCTCTAGGTGTG<br>Rev:GAATCAATTGTAACAGATGGAATTTGAATC
STAT1/P2 mut		Fw:GCCCTGGCTGTACTACCACTCACTCTTTAG<br>Rev:GTGGTAGTACAGCCAGGGCTACACTGAGAA
STAT1/P2 del		Fw:GTAGCCCTGGCTGCTCACTCTTTAGACTAG<br>Rev:GAGTGAGCAGCCAGGGCTACACTGAGAAAC

fw: forward primer, rev: reverse primer, mut: mutation, del: deletion.

Table S3 qPCR primers for gene expression

Gene	Forward primer 5'-3'	Reverse primer 5'-3'
Bcl6	AGACGCACAGTGACAAACCATACAA	GCTCCACAAATGTTACAGCGATAGG
Pref-1	CTTTCTCAACAAGTGCGAAACC	AGTGGTCATGTCAATCTTCTCG
Zfp423	CAAGAAGTCCAAGGCTGAG	TTGAGGTCGCACTGATTG
Zfp521	CTCAGCAGACCTCCGATAT	GTAAGACCTCCAAGCAATACT
Zfp467	CTTATGGCTGCGAGGAATG	GGTGAATCTTCTGGCTGAA
Zfp395	GGAGATGGACGAGATGATG	ACGGGAAACAGAGAAGATG
Ebf1	ACAAGCCACCAATCAAGG	GAAGGAGAAGATGCCAGAG
KLF15	GCCTTCTGTTCCTGCTAC	CCACTGCTATCTCCAATACC
C/EBPδ	AAGAGTAAGACCAAGAAGACC	GCTCCAGGACCTTATGCT
C/EBPα	AGCCAAGAAGTCGGTAGA	CGGTCATTGTCACTGGTC
PPARγ2	TGGGTGAAACTCTGGGAGATTC	AGAGGTCCACAGAGCTGATTCC
PRDM16	AGGATTGCGAGCGGATGTT	GGCGGATGAGGTTGGACTT
UCP1	GGCTTAATGACTGGAGGTGTG	TTCTGTGGTGGCTATAACTCTGT
aP2	GATGCCTTTGTGGGAACCTG	TCCTGTCGTCTGCGGTGATT
LPL	TCTCCTGATGACGCTGATTTTG	TCTCTTGGCTCTGACCTTGTTG
AdipoQ	CCTCTTAATCCTGCCCAGTC	TCCTGTCTCACCCTTAGGAC
STAT1	AGTGGCTGGAAAAGCAAGAC	ATCCTGGAGATTACGCTTGC
ZBTB32	TCAGATGGAGACACATTACC	CGAGGAAGAGTAGAGGAAG
Arrdc3	AAGTGTGAGCAGTCAGTGTAG	GATTATTCCGTCTCTGTTCCTC
Aldh1a	CGTGAACCTATTGGAGTGT	CTCTGCTGGCTTGACAAC
STAT5a	CAACATCAGCAGCAACCA	CTTCTTCAGCACCTCCATC
Id2	CAAGAAGGTGACCAAGATG	CAAGGACAGGATGCTGAT
BMP4	ATCACCTCAACTCAACCAA	CCTCTACCACCATCTCCT
E2F6	AGAACTCTCCGACCTGTC	CTTGAATGCCGTGAATATCC
β-actin	GGCACCACACCTTCTACAATG	GGGGTGTTGAAGGTCTCAAAC

Table S4 PCR primers for shRNA expression vector

shRNA plasmids	Sequence 5'-3'
pRNAT-shBcl6	Fw:gatccGCAGACGCACAGTGACAAATTCAAGAGATTTGTCACTGTGCGTCTGCTTTTTTACGCGTa<br>Rev:agcttACGCGTAAAAAAGCAGACGCACAGTGACAAATCTCTTGAATTTGTCACTGTGCGTCTGCg
pRNAT-shNC	Fw:gatccGTTCTCCGAACGTGTCACGTTTCAAGAGAACGTGACACGTTCGGAGAATTTTTTACGCGTa<br>Rev:agcttACGCGTAAAAAATTCTCCGAACGTGTCACGTTCTCTTGAAACGTGACACGTTCGGAGAACg

fw: forward primer, rev: reverse primer.

Table S5 Searching Bcl6 sites in the targets promoter[1]

Gene symbol	Matrix	Position	Strand	Matrix sim.	Sequence
Stat1	V$BCL6	-2021/-2005	-	0.815	cagtttcTGGAaggaaa
		-1696/-1680	-	0.973	tgtTTCCttgaaacaga
		-1526/-1510	-	0.885	gagTTCCaggacagcca
		-1291/-1275	-	0.819	ttattctTTGAataagc
Nmi	V$BCL6	-2828/-2812	+	0.826	ctgtttcTTGAaaagaa
		-1286/-1270	-	0.91	ttgTTCCgagaaatcag
		-234/-218	-	0.885	gagTTCCaggacagcca
Casz1	V$BCL6	-1515/-1499	-	0.913	ttaTTCCaagaattaac
		-1513/-1497	+	0.828	taattctTGGAataagg
		-1359/-1343	+	0.886	attTTCCtacaagtgtg
		-1279/-1263	-	0.88	gagTTCCtaaataaaac
		-398/-382	+	0.854	attttcaTTGAaaaagt
Ifi44	V$BCL6	-2569/-2553	+	0.886	cagTTCCaggagacatt
		-703/-687	-	0.885	gagTTCCaggacagcca
		-113/-97	+	0.882	tgcTTCCtaaaatttcc
Ifih1	V$BCL6	-2330/-2314	+	0.968	tttTTCCttgaaggggg
		-2295/-2279	+	0.813	agagtctTAGAaaacgt
		-1114/-1098	-	0.875	ggattctTTGAaaacag
Ifit1	V$BCL6	-692/-676	+	0.815	tgattggTGGAaagaac

[1]Indentifying the binding sites of Bcl6 in the targets promoter by MatInspector program available at http://www.genomatix.de/matinspector.html. The promoter sequence of the targets were obtained from genome database (http://asia.ensembl.org/index.html). The 3000 bp upstream of the first exon (in which the first base was defined as +1) of the genes used to search Bcl6 binding sites with a fixed matrix threshold of 0.8.

Table S6 Searching STAT1 sites in the promoter of well-characterized pro-adipogenic genes[1]

Gene symbol	Matrix	Position	Strand	Matrix sim.	Sequence
Zfp423	V$STAT1	-124/-106	+	0.771	aaaaatccgGCAAggggcg
Zfp467	V$STAT1	-2180/-2162	+	0.851	attctccctGGAAacaggc
		-1568/-1550	+	0.883	aaggatccaGGAAgatggg
Zfp395	V$STAT1	-744/-726	+	0.821	ttaattccaGAAAcagtaa
		-2829/-2811	-	0.782	ggctgtcctGGAActcact
Ebf1	V$STAT1	-2032/-2014	-	0.885	tgggtcccaGGAAgcccta
KLF15	V$STAT1	-372/-354	-	0.782	ttactcacgGGAAtggacg
KLF4	V$STAT1	-1485/-1467	-	0.772	ttgagtcccGGAAtccttg
C/EBPδ	V$STAT1	-1640/-1622	+	0.862	acggtgctaGGAAaggtcc
		-2242/-2224	+	0.858	tgaatgctaGGAAgtttta
C/EBPα	V$STAT1	-2990/-2972	+	0.787	gccgctcctGGAAgaggaa
PPARγ	V$STAT1	-468/-450	+	0.862	gcacatctaGGAAaaaaac
		-1476/-1458	-	0.782	ggctgtcctGGAActcact
		-2669/-2651	-	0.853	tgaatgctaGGAActgggc

[1]Indentifying the binding sites of STAT1 in the promoter of well-characterized pro-adipogenic genes by MatInspector program available at http://www.genomatix.de/matinspector.html. The promoter sequence of the pro-adipogenic genes were obtained from genome database (http://asia.ensembl.org/index.html). The 3000 bp upstream of the first exon (in which the first base was defined as +1) of the genes used to search STAT1 binding sites with a default program.
